# Mechanism of Erianin anti-triple negative breast cancer based on transcriptomics methods and network pharmacology

**DOI:** 10.18632/aging.205516

**Published:** 2024-02-07

**Authors:** Ming Li, Yuan Zhao, Huimin Li, Shiyao Kang, Xuming Deng, Miaomiao Sheng

**Affiliations:** 1 Laboratory of Molecular Genetics of Aging and Tumour, Medical School, Kunming University of Science and Technology, Chenggong Campus, Kunming, Yunnan 650500, China; 2 Kunming University of Science and Technology Affiliated Puer City People’s Hospital, Puer, Yunnan 665000, China

**Keywords:** Erianin, triple-negative breast cancer, network pharmacology, transcriptomics, molecular mechanism

## Abstract

Triple negative breast cancer (TNBC) is a highly aggressive illness that lacks effective targeted treatments. Although Erianin has shown potential antitumor properties, its precise mechanism of action and target in TNBC remain unclear, hampering the development of drugs. The present study investigated the underlying mechanism of action of Erianin in treating TNBC by using transcriptomics and network pharmacology approaches. We evaluated Erianin’s bioactivity in TNBC cell lines and xenograft tumor models. The results showed that Erianin significantly inhibited TNBC cell proliferation and impeded tumor growth. A subsequent analysis of transcriptomic and network pharmacological data identified 51 mutual targets. Analysis of protein-protein interactions identified eight hub targets. Furthermore, molecular docking indicated that the PPARA binding energy was the lowest for Erianin among the hub targets, followed by ROCK2, PDGFRB, CCND1, MUC1, and CDK1. Gene Ontology and Kyoto Encyclopedia of Genes and Genomes functional enrichment analysis showed that the common targets were associated with multiple cancer-related signaling pathways, including focal adhesion, PI3K-Akt signaling pathway, Rap1 signaling pathway, microRNAs in cancer, and human papillomavirus infection. The results of the Western blot and immunohistochemistry experiment further showed that Erianin could suppress PI3K/Akt signaling pathway activation. After co-incubation with SC79, the cell inhibition rate of Erianin was decreased, which further confirmed that Erianin inhibits TNBC progression via the PI3K-AKT signaling pathway. In conclusion, our results indicated that Erianin has the potential to inhibit the proliferation of TNBC by downregulating the PI3K/AKT signaling pathway by transcriptomics and network pharmacology. Therefore, Erianin appears to be a promising compound for the effective treatment of TNBC.

## INTRODUCTION

Breast cancer (BC) is the most commonly diagnosed cancer in women, posing a significant threat to women’s health. The statistics on cancer in 2020 reveal that the incidence of BC is remarkably high, with 2.26 million new cases, exceeding the 2.2 million cases of lung cancer, thus making it the most prevalent cancer across the globe [1]. In China, the yearly incidence and mortality rates of BC constitute 12.2% and 9.6% of the global sum, respectively [2]. TNBC is a type of BC, comprising 10% to 20% of all invasive BCs [3]. Because it does not express estrogen receptor (ER), progesterone receptor (PR) or human epidermal growth factor receptor 2 (HER2) and lacks clear therapeutic targets, endocrine therapy and anti-HER2 therapy are often not effective in TNBC patients. At the same time, TNBC exhibits the traits of heightened metastasis and recurrence [4–6]. At present, the combination of taxane and anthracycline is the common choice for the clinical treatment of TNBC. However, anthracycline causes irreversible toxic damage to the heart, which is difficult for many patients to tolerate. Moreover, once patients develop chemotherapy drug resistance, the tumor will relapse and metastasize rapidly [7, 8]. Therefore, research and development of anticancer drugs with high efficiency and low toxicity has become a hot and difficult point in the treatment of TNBC.

Increasing attention is being paid to research on the study and application of substrates derived from natural plants. Erianin (2-methoxy-5-(2-(3,4,5-trimethoxy phenyl) ethyl)-phenol), a bibenzyl compound, is one of the active compounds extracted from Dendrobium. It has numerous biological functions, including programmed cell death induction, angiogenesis inhibition, and antioxidant and antitumor properties. Previous studies have shown that Erianin can inhibit the growth of tumor cells by inducing apoptosis [9, 10], autophagy [11, 12], ferroptosis [13] and other pathways. Sheng et al. [14] found that Erianin could exert its anti-liver cancer effect by inhibiting the activity of pyruvate carboxylase. Chen et al. [13] found that Erianin could inhibit the proliferation and migration of lung cancer cells via calcium/calmodulin-dependent ferroptosis. At present, little is known about the antitumor effects of Erianin on human BC cells in the BC setting, and it has been reported that Erianin can inhibit the proliferation and migration of T47D cells and can also induce cell apoptosis [15]. Erianin and its derivatives (Ecust004) can suppress BC cell growth, invasion, and migration via EMT regulation [16]. Erianin induces apoptosis in TNBC cells by inhibiting the PI3K/Akt pathway [17]. However, the molecular mechanism of action and the drug targets of Erianin in TNBC are unclear, which limits the further development of this natural anticancer product with significant potential.

**Figure 1:**
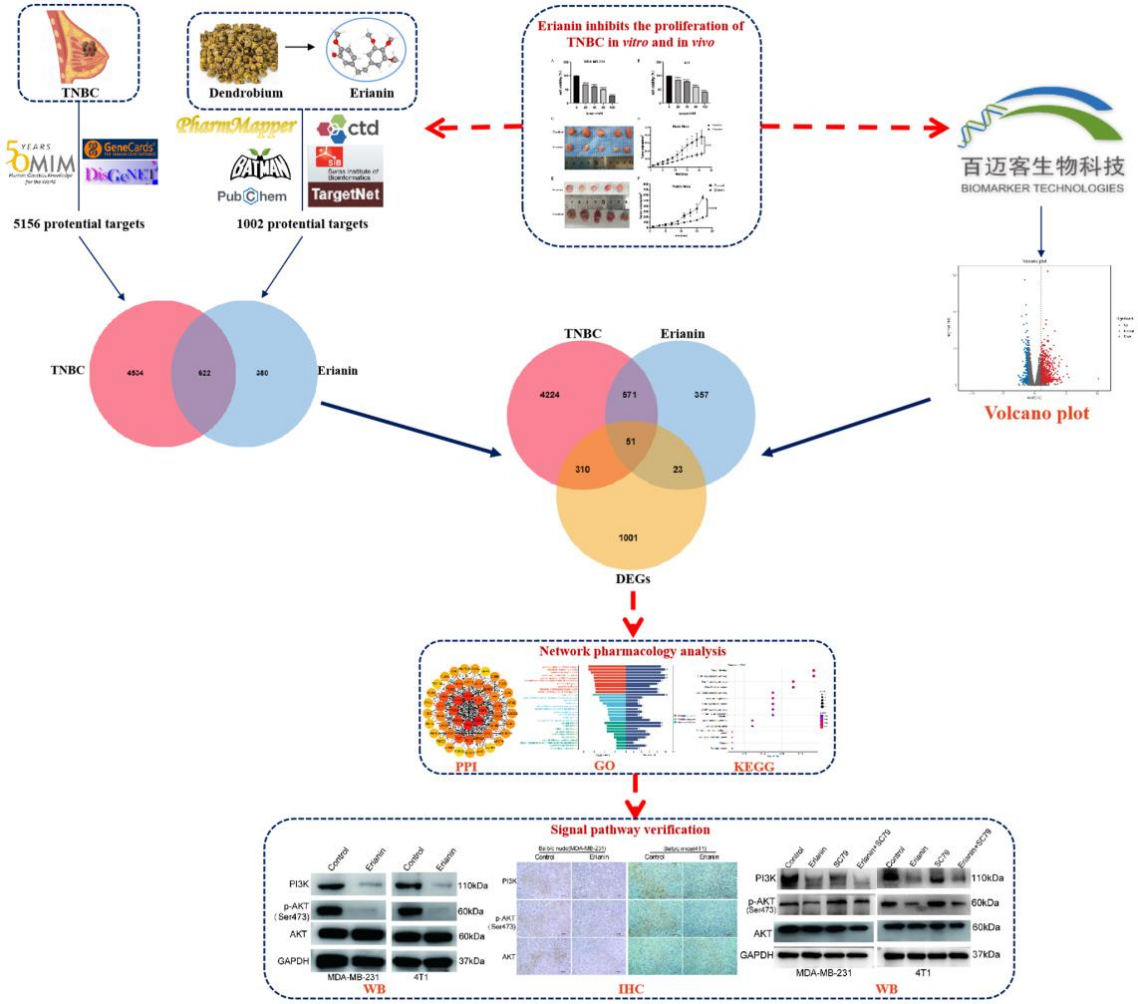
Workflow diagram of the research.

In recent years, network pharmacology has been used to study the pharmacological mechanisms of traditional Chinese medicine. It can mechanically associate drugs and diseases, quantitatively represent the key nodes of the network, including key molecules, key pathways or key modules, and predict the relationship between drugs and disease targets. In this study, we utilized transcriptomics and network pharmacology to explore the antiproliferative mechanism of Erianin in TNBC (Figure 1). First, we evaluated the tumor inhibitory effect of Erianin on TNBC *in vitro* and *in vivo*. Next, common targets, hub genes, biological functions and KEGG signaling pathways of Erianin against TNBC were analyzed by network pharmacology and transcriptomics. Furthermore, molecular docking analysis was used to predict the target genes of Erianin. Finally, the results were validated through biological experiments. Then, the PI3K-AKT signaling pathway was validated.

## MATERIALS AND METHODS

### Cell culture

The human BC cell line MDA-MB-231 and mouse BC cell line 4T1 were purchased from the Peking Union Medical College Cell Culture Center (Beijing, China). MDA-MB-231 cells were cultured in Dulbecco’s modified Eagle’s medium (DMEM; C11995500BT, Thermo Fisher Scientific, USA) supplemented with 10% fetal bovine serum (FBS, 10099141, Thermo Fisher Scientific). The 4T1 cells were maintained in Roswell Park Memorial Institute-1640 medium (C11875500BT, Thermo Fisher Scientific) supplemented with 10% FBS. All cells were cultured in a humidified atmosphere of 5% CO_2_ at 37°C.

### Reagents

Purified Erianin (HPLC ≥98%) was purchased from Shanghai Yuanye Biotechnology Co., Ltd. (Shanghai, China, B20844). A stock solution at 800 μM was made in DMSO (Sigma, USA) and stored in the dark at −20°C. RIPA (P0013B) and modified sodium citrate antigen repair solution (P0083) were purchased from Beyotime Biotechnology (China), Cell Counting Kit-8 (CCK-8) detection kit was purchased from RiboBio (C-005) (China), goat serum was purchased from Jackson (005-000-121) (USA), immunohistochemical secondary antibody and DAB developer were purchased from Dako (K5007) (Denmark), Antibodies to PI3K (4257p), Phospho-Akt (Ser473, 4060S) and AKT (3788S) were purchased from Cell Signaling Technology (USA). GAPDH (AC001) was purchased from ABclonal (USA). SC79 (HY-18749) was purchased from MedChemExpress (USA).

### CCK-8 assay

The CCK-8 experiment was used to evaluate the effect of drug treatment on the proliferation and viability of MDA-MB-231 and 4T1 cells. The cells were inoculated into 48-well plates at a density of 2 × 10^4^ cells per well. When the cells were 70% to 80% confluent, the cells were treated with the drug, and after a certain period of time, 20 μl of CCK-8 solution was added to each well. Then, the optical density (OD) at 450 nm was measured by an enzyme meter. Cell survival rate (%) = (OD experimental group-OD blank group)/(OD negative control group-OD blank group) × 100%.

### Wound healing assay

Cells were seeded (4 × 10^5^ cells/well) in a 6-well plate, when the cell density was above 90%, some straight lines were drawn on the bottom of the 6-well plate with a sterile pipette tip. Discarded the old medium, washed it twice with preheated PBS, and added medium containing 1% serum. Then placed the 6-well plate in the incubator (37°C, 5% CO_2_) 24 hours. After the experiment, ImageJ software was used for data analysis.

### *In vivo* xenograft assay

Six- to eight-week-old female Balb/c normal mice and Balb/c nude mice were purchased from Kunming Medical University. The mice were housed under a specific pathogen-free (SPF)-grade experimental system. Each Balb/c mouse was injected with 3 × 10^5^ 4T1 cells, and each Balb/c nude mouse was injected with 3 × 10^5^ MDA-MB-231 cells in the second left mammary fat pad. When the tumor size reached approximately 15 mm^3^, all mice were randomly divided into two groups: the experimental group and the control group (five mice in each group). The mice in the experimental group were injected with 4 mg/kg Erianin (based on previous studies [9, 12, 18–23] and our experimental validation), while the control group received an injection of the same volume of 1% DMSO. Every two days, the tumor volume was measured. The volume was calculated using the following formula: V = 0.5 × length × width^2^. Nude mice were executed after 21 days and Balb/c mice after 17 days and the tumours were photographed and recorded. All protocols were approved by the Animal Ethics Committee of Kunming University of Science and Technology (PZWH K2019-0005).

### Transcriptome sequencing and analysis

Cells in the logarithmic growth phase were passaged. After 80% cell confluence, one dish was randomly selected as the experimental group and treated with 40 nM of Erianin. Similarly, another dish was randomly selected as the control group and treated with an equivalent volume of solvent (1‰ DMSO). The cells were then collected after 24 hours, and three samples were replicated three times. Subsequently, the cells were sent to Biomarker Technologies for transcriptome sequencing. Cell samples were sent to Bemac Biotechnology Ltd., for transcriptome sequencing. According to the method described by the company, RNA was initially extracted from the samples. RNA purity and concentration were assessed using a NanoDrop 2000 spectrophotometer. After accurate detection of RNA integrity by Agilent 2100/LabChip GX, library construction occurred upon passing the test. Library quality control was carried out employing the Qubit 3.0 fluorescence quantification instrument, Qsep400, and Q-PCR method. PCR methods were utilized for quality control of the library before sequencing using the Illumina NovaSeq 6000 sequencing platform. The differentially expressed genes (DEGs) were screened for expression differences using the condition | log2Fold Change | >1.

### Drug and disease target acquisition

Exploring the genes associated with targeting a certain disease is an important foundation in modern small molecule drug discovery and development. To explore the genes associated with the anti-TNBC progression of Erianin, we analyzed the target genes of Erianin against TNBC by using web-based pharmacology. First, we obtained the 2D structure and 3D structure of Erianin from PubChem through BATMAN-TCM [24] (http://bionet.ncpsb.org.cn/batman-tcm/index.php), PharmMapper [25] (http://www.lilab-ecust.cn/pharmmapper/), Swiss Target Prediction (http://swisstargetprediction.ch/), TargetNet [26] (http://targetnet.scbdd.com/), PubChem (https://pubchem.ncbi.nlm.nih.gov/) and CTD [27] (https://ctdbase.org/) databases to obtain the targets of action of Erianin. Where the BATMAN-TCM database was obtained for Erianin-related targets and screened for “Score cutoff >2.608” targets. Through the UniProt database, target names were standardized, and duplicates were removed and merged to create the Erianin target dataset. “Triple negative breast cancer” or “TNBC” were used as the search terms, and the data were obtained from the GeneCards (https://www.genecards.org/), OMIM (https://www.omim.org/) and DisGenet [28] (https://www.disgenet.org/) databases. Among them, TNBC-related gene information was retrieved from the GeneCards database, and the genes with GeneCards GIFtS greater than 40 were screened. Subsequently, target names were standardized, and duplicates were removed and merged to establish the TNBC-related gene dataset through the UniProt database.

### Construction of the protein-protein interaction and hub gene network

The online network analysis platform, STRING 11.5 [29] (http://string-db.org/) was used to analyze the network topology. The common targets were inputted into the STRING 11.5 database for creation, with the selection of “*Homo sapiens*” as the species. Network was then visualized and analyzed using Cytoscape 3.8.0. The Analyze Network and CytaHubba were then used to screen the hub genes based on degree in the PPI network.

### Molecular docking analysis

Molecular docking can effectively predict the binding abilities between small molecule compounds and target genes. The 2D structure of Erianin was downloaded from the PubChem database. The 2D structure was then converted to a 3D structure in Chem3D software. The 3D crystal structures of 8 hub genes were retrieved from the PDB database. AutoDockTools 1.5.6 software was utilized to remove all ligands from the protein receptors and add hydrogens and charges before molecular docking simulation. Ligands and protein receptors were recorded in PDBQT format. Then, the binding sites of Erianin to the target gene receptor protein were examined using PyMOL 2.4.1. The binding energy was determined from the affinity. The higher the absolute affinity value, the stronger the binding affinity of Erianin with proteins.

### Gene expression and survival analysis

The BC gene expression dataset, comprising tumor tissue and paraneoplastic normal tumor samples, was procured from the TCGA database, along with patient-related clinical data. Patients were screened based on clinical information to obtain cases of TNBC with PR-negative, ER-negative, and Her-2-negative conditions. R software was utilized to analyze the expression matrices of both the normal and tumor groups. R software was utilized to extract expression data of potential target genes of Erianin acting on TNBC in the TCGA database. The edge R and DEGseq packages were employed for analyzing and screening DEGs in TNBC tissue samples and normal tissue samples. Histograms were plotted with loglFCl >1 and *p* < 0.05 thresholds. Additionally, we performed a survival analysis on possible target genes of Erianin in TNBC using the Kaplan-Meier Plotter web platform. We selected the best nodes while setting negative ER, PR, and Her-2 statuses. We regarded the genes with *p* < 0.05 in the results as influencing TNBC patient survival.

### Gene function and pathway enrichment analysis

Gene Ontology (GO) [30] is internationally used to analyze the functional enrichment of genes and proteins, while Kyoto Encyclopedia of Genes and Genomes (KEGG) [31] pathway enrichment analysis provides a deeper explanation of different gene and protein functions. We used the R 4.3.1 programming language packages “clusterProfiler”, “org.Hs.eg.db”, “enrichplot” and “ggplot2” to analyze the GO and KEGG enrichment of target genes. The top 10 biological processes (BPs), cellular localizations (CCs) and molecular functions (MFs) in GO analysis and the top 15 pathways in KEGG analysis were screened at *p* < 0.05.

### Western blotting (WB)

BC cells were cultured in 6-well plates at a density of 1 × 10^6^/ml per well. After treatment with Erianin for 24 hours, cells were lysed in precooled RIPA buffer. The protein concentrations were determined by a BCA protein assay kit (Beyotime, P0009). Equal amounts of protein were separated by SDS-PAGE and transferred to 0.45 μm PVDF membranes (Millipore, IPVH00010). After blocking with 2% BSA for 2 hours at room temperature, the membranes were incubated with primary antibodies at 4°C overnight. Then, the membranes were washed with TBST buffer 3 times and incubated with HRP-conjugated secondary antibody. The results were developed and recorded by a chemiluminescence analysis system (Tanon-5200, Tanon Science and Technology, China).

### Immunohistochemistry (IHC)

Tumors were fixed for 24 hours in 4% paraformaldehyde and rinsed in running water. Then, paraffin-embedded tissue was cut into 5 μm thick sections. Sodium citrate buffer was used to boil the slides for 10 minutes to retrieve the antigens. After blocking with goat serum solution for 30 min, the sections were incubated overnight at 4°C with primary antibodies. A secondary antibody was added the next day, DAB chromogenic reagent was added, and the sections were counterstained with hematoxylin. Scores were assigned based on the intensity of the immunohistochemical signal and the area of the tissue sections stained positively. Finally, Image-Pro Plus 6.0 was used for quantitative analysis of the picture.

### Statistical processing

All statistical data were analyzed using GraphPad Prism 8.0 software and R 4.3.1. The data were first tested for normal distribution, followed differences between the two groups were measured by *T*-test, and comparisons differences between the two groups were evaluated by one-way analysis of variance (ANOVA). The experimental data are expressed as the mean ± SD. Statistical significance was defined as *p* < 0.05. ^*^p < 0.05, ^***^p < 0.001, ^****^p < 0.0001.

### Data availability statement

The data that support the findings of this study are available on request from the corresponding author, upon reasonable request.

## RESULTS

### Erianin inhibits the progression of TNBC cells

**Figure 2:**
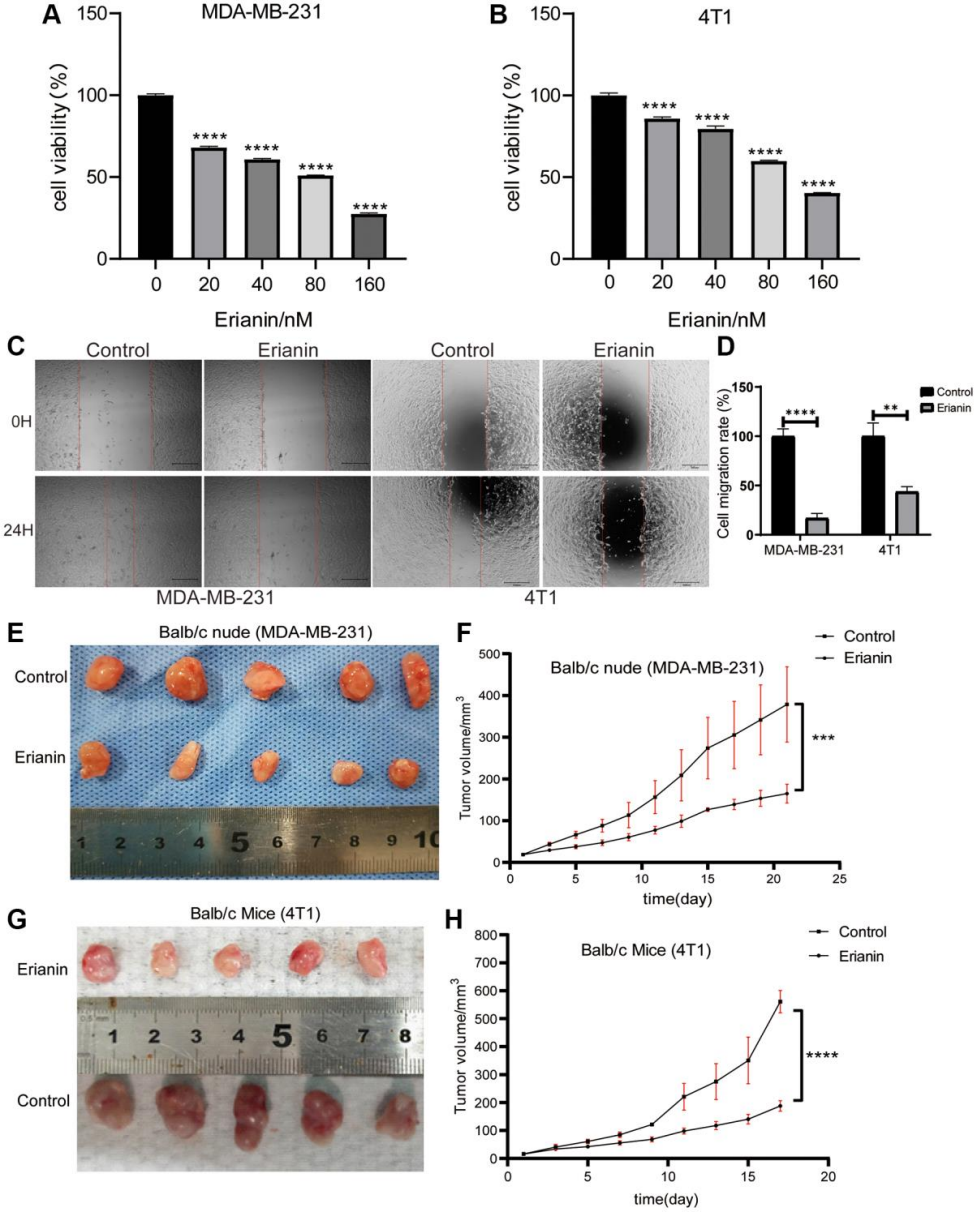
**Erianin inhibits the progression of TNBC cells *in vitro* and *in vivo*.** MDA-MB-231 (**A**) and 4T1 cells (**B**) were treated with different concentrations of Erianin for 24 hours, and then the viability was determined using the CCK-8 assay. (**C**) The migration capacity of MDA-MB-231 and 4T1 cell with Erianin was detected by wound-healing assay. (**D**) Statistical analysis of wound-healing assay. (**E**) MDA-MB-231 cells were injected into Balb/c nude mice *in situ*, and xenograft tumors were imaged after Erianin treatment. (**F**) Tumor volume in each group (*N* = 5). (**G**) 4T1 cells were injected into Balb/c normal mice *in situ*, and xenograft tumors were imaged after Erianin treatment. (**H**) Tumor volume in each group (*N* = 5).

To investigate the antiproliferation of Erianin in TNBC cells, MDA-MB-231 cells and 4T1 cells were treated with different concentrations of Erianin (0, 20, 40, 80, 120, 160 nM) for 24 hours, and the viability of the cells was measured by CCK-8 assay. The results indicated that Erianin had a significant inhibitory effect on the proliferation of MDA-MB-231 and 4T1 cells in a dose-dependent manner (Figure 2A, 2B). Furthermore, we evaluated the effect of Erianin on cell migration. Considering the killing effect of Erianin on TNBC cells, we used 10 nM and 16 nM Erianin to treat MDA-MB-231 and 4T1 cells, respectively. In wound healing assay, Erianin significantly inhibited the migratory ability of MDA-MB-231 and 4T1 cells, compared with the control group (Figure 2C, 2D). In addition, two xenograft transplantation models were used to investigate the antitumor effect of Erianin *in vivo*. MDA-MB-231 cells and 4T1 cells were injected into Balb/c nude mice and Balb/c mice *in situ*. When the tumor size reached approximately 15 mm^3^, the mice were separated randomly into two experimental groups: the control group (DMSO) and the Erianin group. We found that 4 mg/kg Erianin treatment significantly inhibited the tumours growth of MDA-MB-231 and 4T1 cells (Figure 2E–2H).

### Transcriptomic sequencing analysis of TNBC cells after Erianin treatment

**Figure 3:**
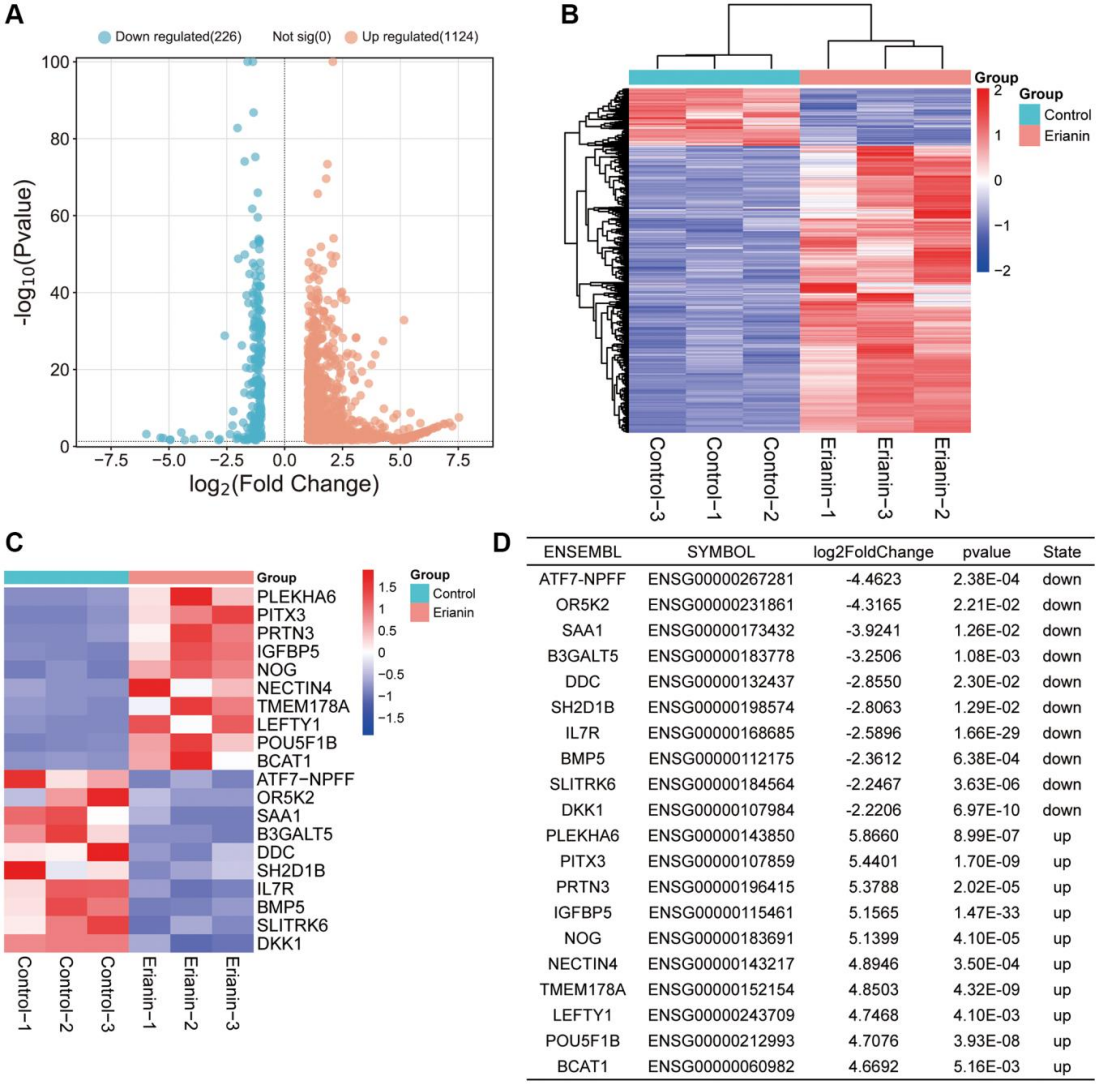
**RNA-seq data analysis.** (**A**) A volcano plot of 1350 DEGs, including 226 downregulated genes and 1124 upregulated genes in response to Erianin treatment, was drawn. (**B**) Heatmap of 1350 DEGs was created. (**C**) Heatmap of the top 10 upregulated or downregulated genes based on the ranking of expression changes. (**D**) Table of the top 10 upregulated or downregulated genes based on the ranking of expression changes.

By comparing the gene expression profile data of the Erianin group and Control group, we identified 1350 DEGs out of 13629 transcriptome annotated genes, following the threshold standard of |log2-fold change| > 1 and *p* < 0.05. The analysis revealed 1124 upregulated genes and 226 downregulated genes. The volcano plot and heatmap of all DEGs were shown in Figure 3A, 3B. To facilitate analysis, we excluded genes that were not expressed in any group from the transcriptome sequencing expression matrix. The top 10 upregulated and downregulated genes were shown in Figure 3C, 3D.

### Network pharmacological screening of Erianin anti-TNBC target genes

**Figure 4:**
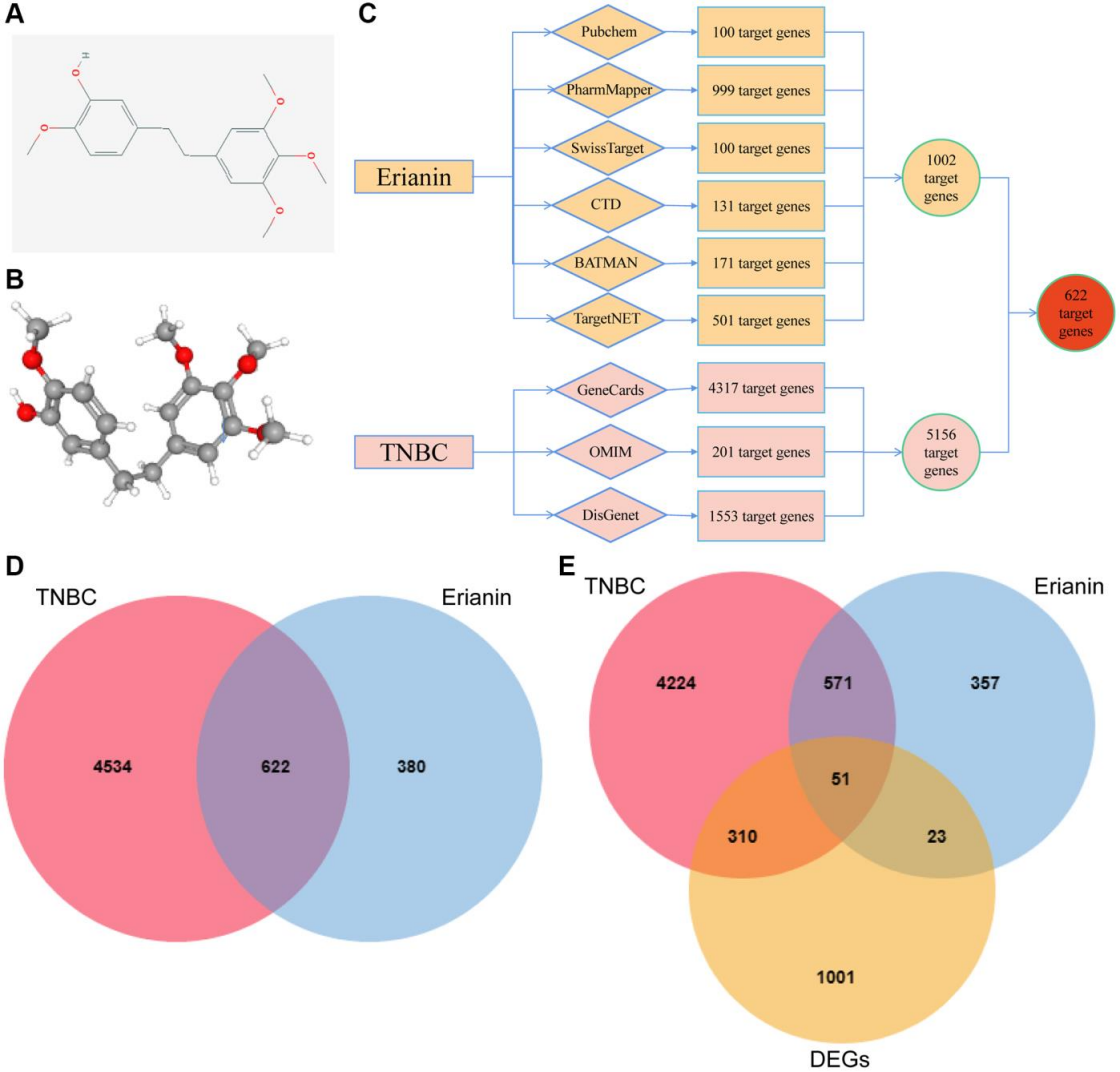
**Screening of target genes for anti-TNBC activity of Erianin.** (**A**) 2D structure of the Erianin molecule. (**B**) 3D structure of the Erianin molecule; (**C**) Flowchart of the screening process of target genes of Erianin anti-TNBC obtained by network pharmacological analysis. (**D**) Venn diagram of target genes of Erianin in anti-TNBC obtained by network pharmacological analysis; (**E**) Venn diagram of target genes identified in network pharmacology analysis and differentially expressed genes (DEGs).

We analyzed the target genes of Erianin against TNBC by using network pharmacology. First, we obtained the 2D structure and 3D structure of Erianin from PubChem (Figure 4A, 4B). Then, we obtained a total of 1002 Erianin-related targets from six drug databases and 5156 TNBC-related targets from the GeneCards, OMIM and DisGenet databases (Figure 4C), and the intersection of these two databases led to the identification of 622 Erianin targets in the TNBC-related target genes (Figure 4D). Furthermore, we combined the potential target genes obtained from the network pharmacological analysis and the differential genes analyzed by transcriptome sequencing, and a total of 51 intersections were obtained (Figure 4E). We carried out subsequent analyses of these 51 common targets.

### PPI network analysis of common targets

**Figure 5:**
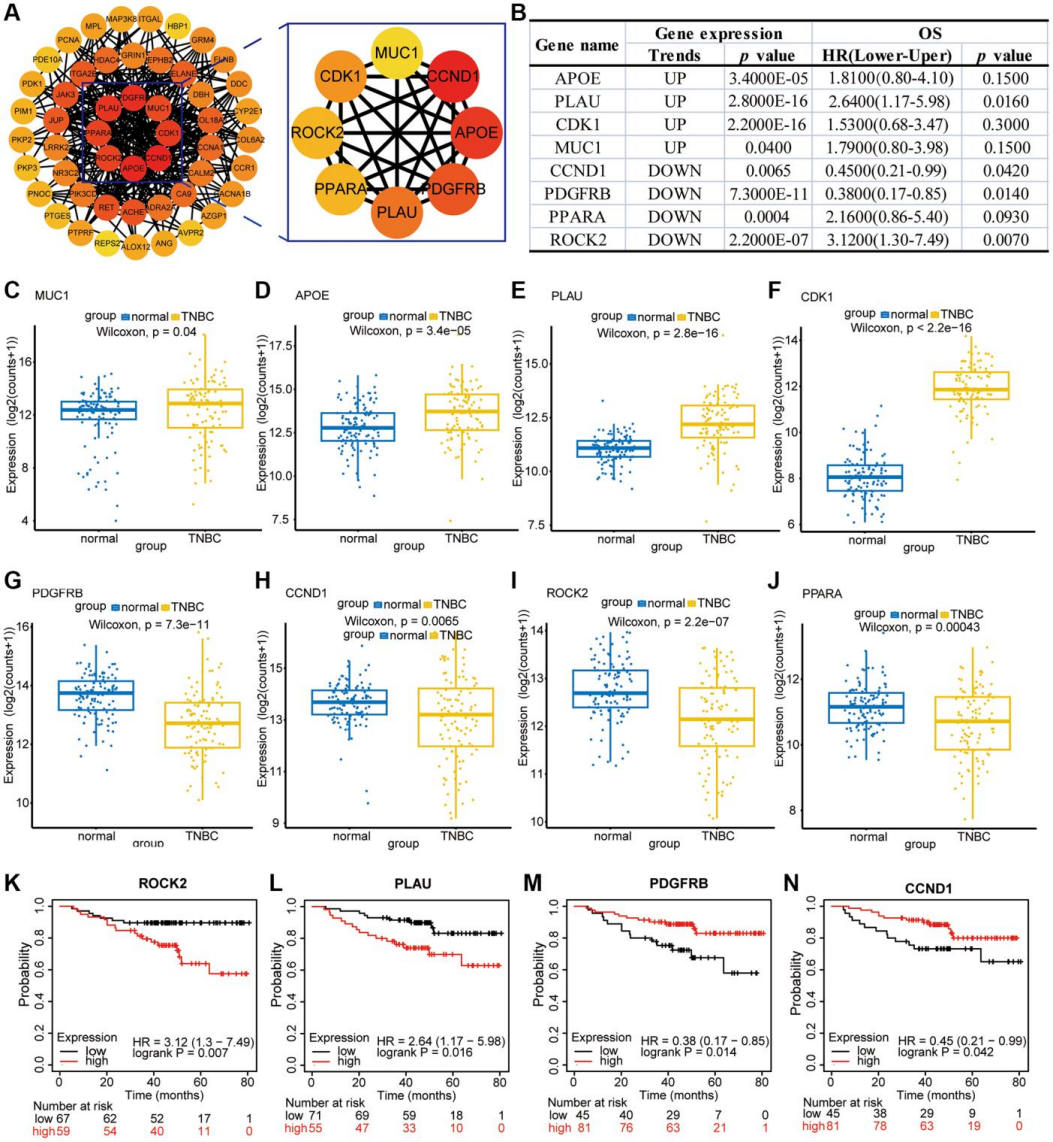
**Bioinformatic analysis of PPI-enriched and hub genes.** (**A**) PPI of potential hub target genes. (**B**) The expression of hub genes in TNBC and the correlation analysis with the overall survival time of patients. (**C**–**J**) Expression maps of MUC1, APOE, PLAU, CDK1, PDGFRB, CCND1, ROCK2, and PPARA in TNBC patients and normal tissues. (**K**–**N**) Impact of ROCK2, PLAU, PDGFRB, and CCND1 expression on the survival of TNBC patients.

The clarity of drug targets is crucial for the in-depth development of drugs, and although our preliminary studies have shown that Erianin plays an important regulatory role in anti-TNBC progression, its target of action in TNBC has not yet been reported in the literature. Therefore, using the STRING database, we explored the interactions between the 51 common targets obtained from the above transcriptomics and network pharmacology to mine the interactions, selecting a confidence score >0.15. A complete interaction network was constructed using Cytoscape, and a total of 51 nodes and 314 edges were obtained. The darker the color, the higher the corresponding degree value. In the PPI network, we employed the degree method to identify the top 8 hub genes. These include CCND1, APOE, PDGFRB, PLAU, CDK1, PPARA, ROCK2 and MUC1 (Figure 5A). Most of these proteins had significant expression differences in TNBC patients (Figure 5B). For example, APOE, PLAU, CDK1 and MUC1 were significantly higher in TNBC tissues than in cancer-parasite tissues, whereas PDGFRB, RET, ROCK2, CCND1 and PPARA were significantly lower in TNBC tissues than in cancer-parasite tissues (Figure 5C–5J). In addition, high expression of ROCK2 and PLAU in TNBC patients predicted poorer survival, whereas low expression of PDGFRB and CCND1 in TNBC patients predicted poorer survival (Figure 5K–5N).

**Figure 6:**
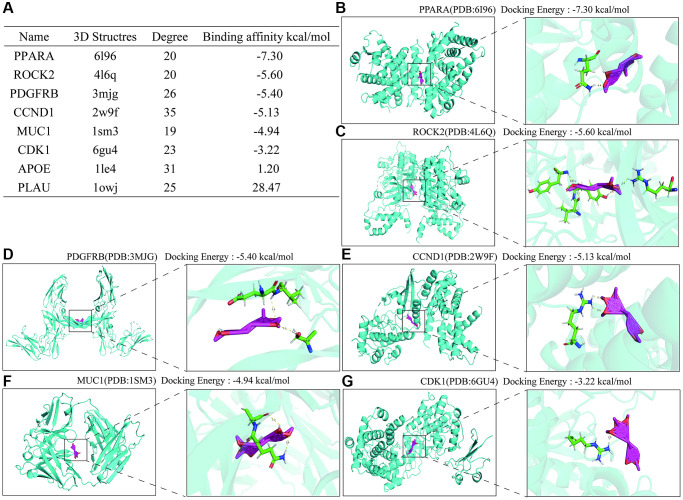
**Molecular docking results of Hub protein and Erianin.** (**A**) Table of molecular docking results of Erianin and hub genes. (**B**–**G**) The molecular docking visualization results of Erianin binding to PPARA, ROCK2, PDGFRB, CCND1, MUC1 and CDK1.

To further clarify the specific targets of Erianin anti-TNBC, we performed molecular docking of these hub genes as target targets with Erianin, and the specific docking binding results were shown in Figure 6A. As shown in the table, Erianin has the lowest binding energy to PPARA, followed by ROCK2, PDGFRB, CCND1, MUC1 and CDK1. The lower docking binding energy indicates that Erianin has a higher binding affinity to these targets, suggesting that it has a greater possibility to act on these targets, affecting the structural changes of the targets and then regulating the corresponding signaling pathways. Docking structures of Erianin with the target proteins PPARA, ROCK2, PDGFRB, CCND1, MUC1 and CDK1 were shown in Figure 6B–6G.

### Functional and pathway enrichment analyses of common targets

**Figure 7:**
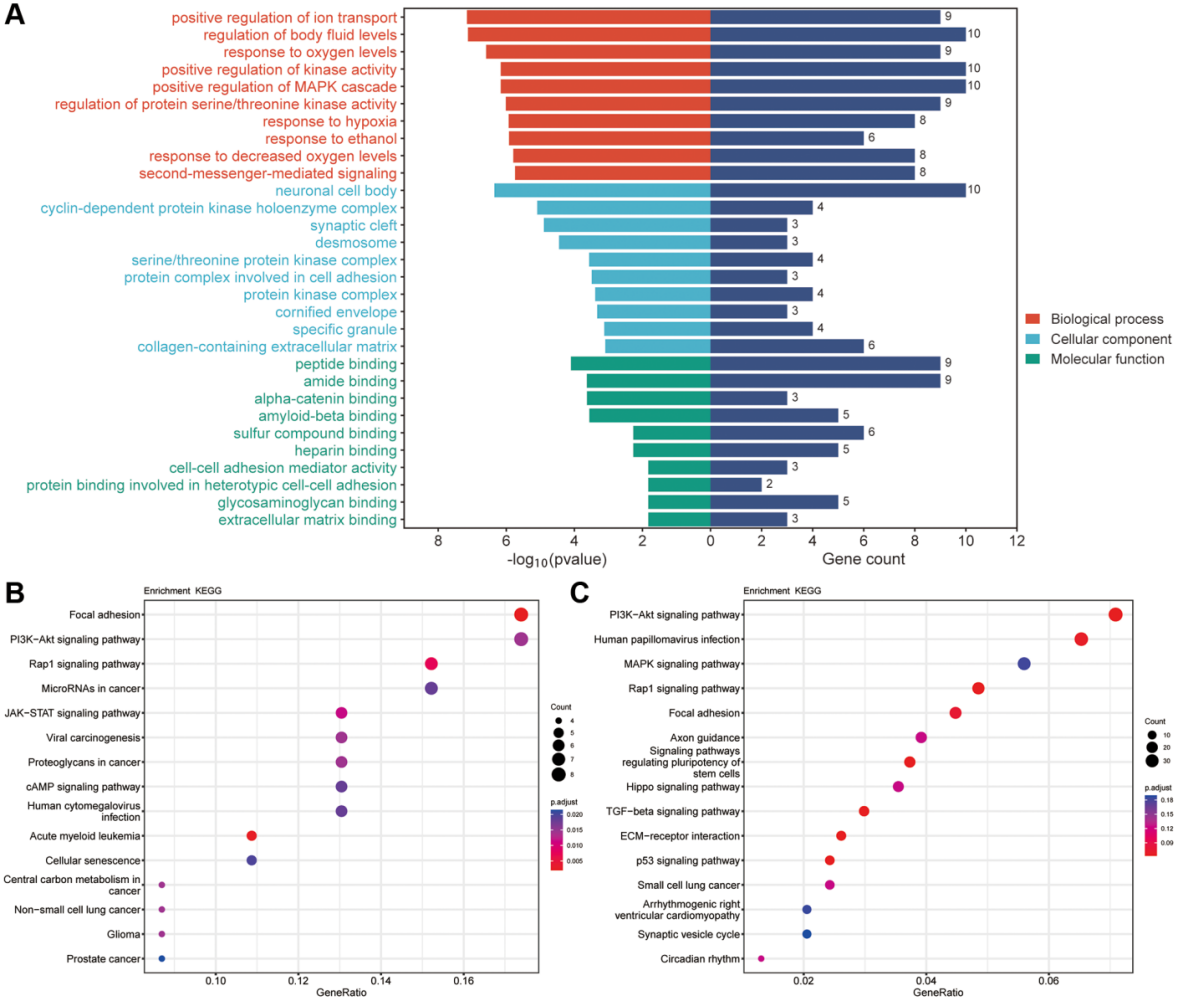
**The results of GO and KEGG pathway enrichment.** (**A**) Top 10 results of GO for target genes of Erianin against TNBC. (**B**) Top 15 enriched KEGG pathways of target genes of Erianin against TNBC. (**C**) KEGG results for DEGs obtained by transcriptome sequencing.

Furthermore, we analyzed GO with KEGG enrichment for 51 common targets and found that there were 2427 results related to BPs, of which 1007 results were significantly different (*p* < 0.05), 220 results related to CCs, of which 72 results were significantly different (*p* < 0.05), and 298 results related to MFs, of which 95 results were significantly different (*p* < 0.05). The GO results are shown in Figure 7A (TOP10). In terms of BP, these genes were enriched in positive regulation of ion transport, regulation of body fluid levels, response to oxygen levels, positive regulation of kinase activity and positive regulation of MAPK cascade. CCs alterations were mainly involved in neuronal cell bodies, cyclin-dependent protein kinase holoenzyme complexes, synaptic clefts, desmosomes and serine/threonine protein kinase complexes. The MFs included peptide binding, amide binding, alpha-catenin binding, amyloid-beta binding and sulfur compound binding. The KEGG results showed that these potential target genes were enriched in a total of 206 pathways, of which 60 pathways were significantly different (*p* < 0.05), including focal adhesion, the PI3K-Akt signaling pathway, the Rap1 signaling pathway, microRNAs in cancer and human papillomavirus infection (Figure 7B). Among them, focal adhesion and the PI3K-AKT signaling pathway were significantly enriched. In addition, we conducted KEGG enrichment analysis on the differentially expressed genes (DEGs) obtained from transcriptome sequencing and found that the PI3K-AKT signaling pathway was significantly enriched among the DEGs (Figure 7C).

**Figure 8:**
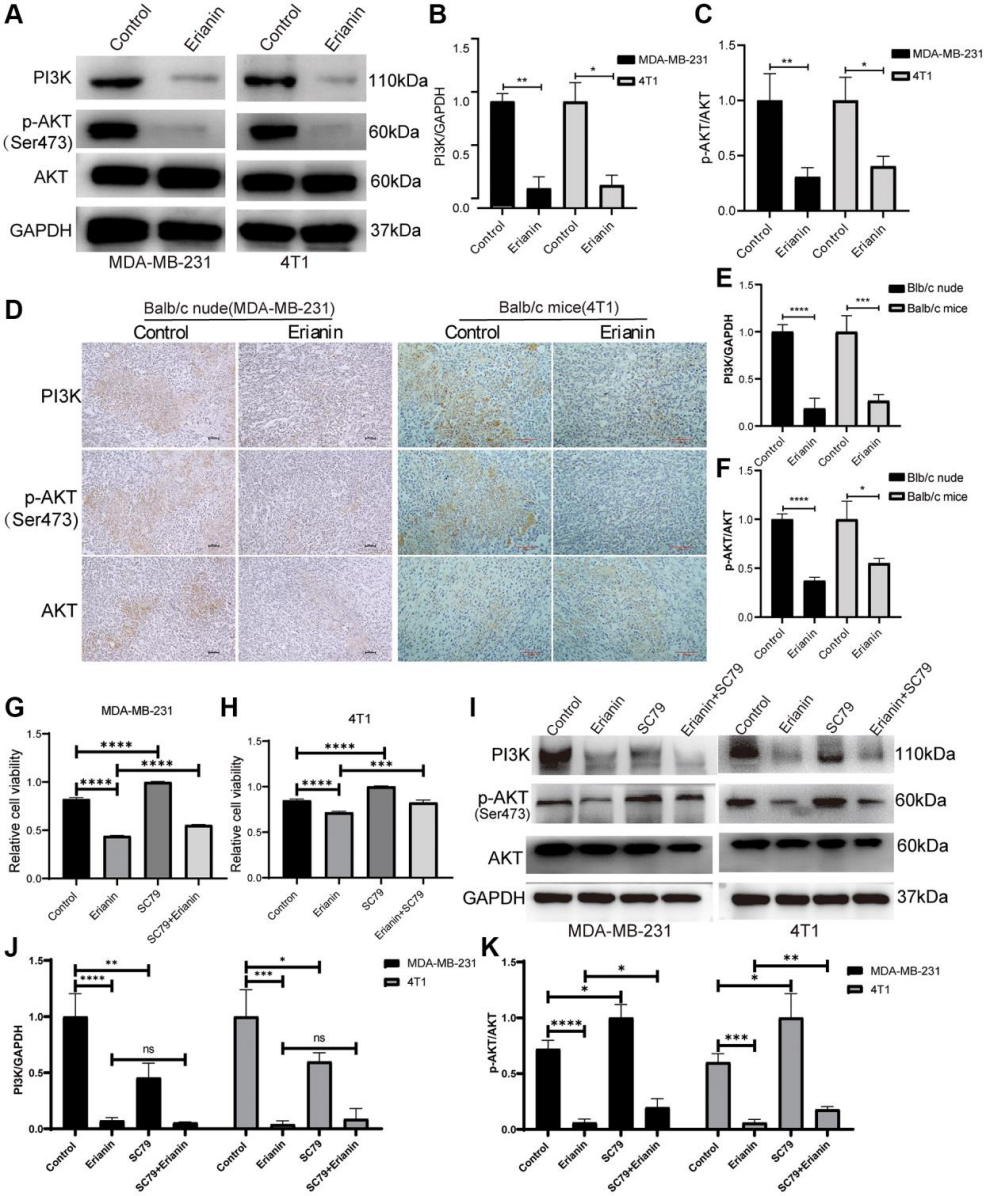
**Erianin inhibits the PI3K-AKT signaling pathway in TNBC.** (**A**) Western blot detection of PI3K and AKT expression as well as AKT phosphorylation after Erianin treatment of MDA-MB-231 and 4T1 cells. (**B**) Quantitative graphs of PI3K expression after Erianin treatment of MDA-MB-231 and 4T1 cells. (**C**) Quantitative graphs of p-AKT expression after Erianin treatment of MDA-MB-231 and 4T1 cells. (**D**) Immunohistochemistry detection of PI3K and AKT expression as well as AKT phosphorylation in two transplantation tumor models. (**E**) Quantitative graphs of PI3K expression after Erianin treatment of two transplantation tumor models. (**F**) Immunohistochemical detection of PI3K and AKT expression as well as AKT phosphorylation in two transplantation tumor models. (**G**) MDA-MB-21 cells were treated with 40 nM of Erianin and 2 ug/ml of SC79 for 24 hours, and then the viability was determined using the CCK-8 assay. (**H**) 4T1 cells were treated with 80 nM of Erianin and 2 ug/ml of SC79 for 24 hours, and then the viability was determined using the CCK-8 assay. (**I**) Western blot detection of AKT expression and AKT phosphorylation after Erianin and SC79 treatment of MDA-MB-231 and 4T1 cells. (**J**) Quantitative graphs of PI3K expression in MDA-MB-231 and 4T1 cells. (**K**) Quantitative graphs of AKT phosphorylation expression in MDA-MB-231 and 4T1 cells.

The PI3K-AKT signaling pathway holds significance in responding to extracellular signals and regulating biological processes, including cell proliferation, apoptosis, and migration. Therefore, we explored the regulatory effects of Erianin on the PI3K-AKT signaling pathway by WB and immunohistochemical experiments. We treated two types of TNBC cells with concentrations of Erianin at 40 nM and 80 nM, respectively, which correspond to the half maximal inhibitory concentration for each cell type. Western blot results showed that Erianin significantly inhibited the expression of PI3K proteins and the phosphorylation of AKT after treating MDA-MB-231 and 4T1 cells for 24 hours (Figure 8A–8C). Furthermore, the expression of key proteins in the PI3K-AKT signaling pathway was also detected by immunohistochemistry in both transplantation tumor models, and the results were consistent with those of Western blot analysis (Figure 8D–8F). To further investigate whether Erianin can regulate the progression of TNBC through the PI3K-AKT signaling pathway, rescue experiments were conducted. We incubated TNBC cells with the Akt agonist SC79, CCK-8 assay revealed that 2 ug/ml SC79 treatment significantly reduced the inhibitory effect of Erianin on the proliferation of MDA-MB-231 and 4T1 cells, suggesting that the activation of the PI3K/Akt pathway was associated with the antitumor effect of Erianin (Figure 8G, 8H). Western blot experiments also showed that SC79 significantly enhanced AKT phosphorylation and attenuated the inhibitory effect of Erianin on AKT phosphorylation (Figure 8I–8K). The above results indicated that Erianin may inhibit TNBC cell proliferation by affecting the PI3K-AKT pathway, which confirmed the findings of network pharmacology and transcriptome sequencing.

## DISCUSSION

TNBC, as a complex heterogeneous disease, does not express ER, PR and HER2 and is characterized by early onset, high invasiveness, poor prognosis, early local recurrence and distant metastasis. TNBC has higher mortality and shorter median survival than non-TNBC. Surgery combined with chemotherapy and radiotherapy is the main treatment for this kind of cancer, and effective targeted therapy strategies are urgently needed.

Compared with conventional chemotherapy, natural products have obvious advantages. They can interact with multiple targets and have high therapeutic value and low systemic toxicity. As a new anticancer drug, they have attracted widespread attention. Erianin is a small molecular dibenzyl compound extracted from *Dendrobium*. Previous studies have demonstrated that Erianin exerts a variety of pharmacological effects, including antiviral [32], antiaging, antidiabetic, and anti-inflammatory activities [33] and inhibits angiogenesis [34, 35]. Especially in the treatment of tumors, it has significant antitumor activity, including liver cancer [10, 36], lung cancer [13, 18, 34], nasopharyngeal cancer [37], cervical cancer [38], bladder cancer [9], osteosarcoma [12], etc., However, the molecular mechanism and cellular targets through which Erianin acts against TNBC are poorly systematically defined, which is important for understanding its potential anticancer mechanisms. In this study, we elucidated the effects of Erianin on the proliferation of TNBC, verified the mechanism predicted by transcriptome analysis and network pharmacology, and explored the potential target of Erianin both *in vitro* and *in vivo*.

First, MDA-MB-231 and 4T1 cells were selected to determine the anti-proliferative activity of Erianin by CKK-8. The results showed that Erianin significantly inhibited the proliferation of TNBC cells in a dose-dependent manner. Meanwhile, Erianin could significantly inhibit the migratory ability of MDA-MB-231 and 4T1 cells, respectively. Further *in vivo* experiments also showed that Erianin treatment notably suppressed the growth of transplanted tumors in mice, indicating that Erianin had strong anti-TNBC proliferation activity. Next, 1385 DEGs were obtained by transcriptome sequencing analysis, and volcano and heatmaps were drawn.

Meanwhile, to explore the genes related to Erianin anti-TNBC progression, we analyzed the target genes of Erianin anti-TNBC using network pharmacology and identified 622 genes shared by Erianin and TNBC. Furthermore, we combined the potential target genes obtained from the network pharmacology analysis and the differential genes analyzed by transcriptome sequencing, and a total of 51 intersections were obtained. To investigate the molecular mechanism and potential targets of Erianin in inhibiting the proliferation of TNBC. We constructed a PPI network on these 51 genes. PPI network analysis screened eight central targets, including CCND1, APOE, PDGFRB, PLAU, CDK1, PPARA, ROCK2 and MUC1. In previous literature, most of these genes have been associated with the onset and progression of TNBC. For example, CCND1 played a significant role in TNBC proliferation, migration, and treatment [39–41]. PDGFRB was found to be significantly upregulated in aggressive TNBC tumor cells and the tumor microenvironment. Inhibition of its expression has been shown to effectively reduce tumor growth and lung metastasis [42, 43]. The cell cycle protein CDK1 was overexpressed in TNBC and may serve as a novel biomarker [41, 44] and therapeutic target for the diagnosis and treatment of TNBC [45]. The PPARA pathway exhibited downregulation in TNBC. Targeting this pathway could serve as a therapeutic strategy for treating TNBC [46]. MUC1 was expressed in TNBC cell lines as a metabolic regulator in TNBC and promotes metabolic reprogramming of glutamine utilization that affects TNBC tumor growth [47]. Targeting tumor MUC1 glycoprotein inhibited the growth of TNBC [48], among others. Meanwhile, these proteins have significant expression differences in TNBC patients; for example, APOE, PLAU, CDK1 and MUC1 were significantly higher in TNBC patients than in paraneoplastic tissues, while PDGFRB, RET, ROCK2, CCND1 and PPARA were significantly lower in TNBC patients than in paraneoplastic tissues. In addition, high expression of ROCK2 and PLAU in TNBC patients predicted poorer survival, while low expression of PDGFRB and CCND1 in TNBC patients predicted poorer survival.

To specify the potential targets of Erianin for TNBC, we performed docking of these hub genes with Erianin. Our results indicated that PPARA had the lowest binding energy to Erianin, followed by ROCK2, PDGFRB, CCND1, MUC1, and CDK1. The lower docking binding energy implies that Erianin exhibits higher binding affinity toward these targets, indicating its greater potential to act upon and affect the target structure, ultimately regulating the corresponding signaling pathways. The PPARA pathwaywas suppressed in breast cancer expression, specifically in subtypes of TNBC [46, 49]. It played a vital role in the invasion and metastasis of TNBC [50, 51]. PPAR-α can also serve as a target for TNBC treatment [52, 53]. A case in point was fenofibrate, a PPAR-α agonist that can induce apoptosis in TNBC cells by activating the NF-κB pathway [53].

Further KEGG enrichment analyses showed that the 51 potential targets may exert antitumor effects by modulating multiple signaling pathways, such as focal adhesion, the PI3K-Akt signaling pathway, the Rap1 signaling pathway, microRNAs in cancer and human papillomavirus infection. Notably, focal adhesion and the PI3K-Akt signaling pathway were significantly enriched. Meanwhile, transcriptome analysis results suggested that the PI3K-Akt signaling pathway may be crucial for cell death induced by Erianin, indicating the significant role of the PI3K-AKT signaling pathway in Erianin anti-TNBC progression. The PI3K/AKT signaling pathway is a crucial pathway that reacts to external signals and facilitates metabolism, proliferation, cell viability, enlargement and angiogenesis. It is considered a classic pathway related to various cancers [54]. Previous studies have shown that Erianin can inhibit the proliferation of BC [17], gastric cancer [55], lung cancer [37], liver cancer [10] and other tumor cells by inhibiting the PI3K/Akt signaling pathway. Therefore, we detected the activity of the PI3K/AKT signaling pathway after Erianin treatment in TNBC. Consistent with the above studies, the expression levels of PI3K and p-AKT were significantly decreased following treatment with Erianin both *in vivo* and *in vitro*. In the rescue experiment, the inhibitory effect of Erianin on AKT phosphorylation and TNBC proliferation was significantly reduced by SC79, an AKT phosphorylation activator. Thus, the PI3K/AKT signaling pathway may play an important role in the growth inhibition of TNBC cells and tissues induced by Erianin.

In general, our study utilized network pharmacology, molecular docking, transcriptome analysis, and *in vitro* and *in vivo* experiments to comprehensively investigate the mechanism by which Erianin can treat TNBC. Our research indicates that Erianin can potentially serve as a viable medication for treating TNBC. This is due to its ability to restrain the growth of TNBC by inhibiting the PI3K/AKT signaling pathway. Meanwhile, it offered a new viewpoint for examining the function of natural small molecular compounds in the therapy of malignant tumors.

## References

[1] Sung H, Ferlay J, Siegel RL, Laversanne M, Soerjomataram I, Jemal A, Bray F. Global Cancer Statistics 2020: GLOBOCAN Estimates of Incidence and Mortality Worldwide for 36 Cancers in 185 Countries. CA Cancer J Clin. 2021; 71:209–49. 10.3322/caac.21660 33538338

[2] Siegel RL, Miller KD, Fuchs HE, Jemal A. Cancer Statistics, 2021. CA Cancer J Clin. 2021; 71:7–33. 10.3322/caac.21654 33433946

[3] Kumar P, Aggarwal R. An overview of triple-negative breast cancer. Arch Gynecol Obstet. 2016; 293:247–69. 10.1007/s00404-015-3859-y 26341644

[4] Lee KL, Kuo YC, Ho YS, Huang YH. Triple-Negative Breast Cancer: Current Understanding and Future Therapeutic Breakthrough Targeting Cancer Stemness. Cancers (Basel). 2019; 11:1334. 10.3390/cancers11091334 31505803 PMC6769912

[5] Garrido-Castro AC, Lin NU, Polyak K. Insights into Molecular Classifications of Triple-Negative Breast Cancer: Improving Patient Selection for Treatment. Cancer Discov. 2019; 9:176–98. 10.1158/2159-8290.CD-18-1177 30679171 PMC6387871

[6] Geiger S, Cnossen JA, Horster S, DiGioia D, Heinemann V, Stemmler HJ. Long-term follow-up of patients with metastatic breast cancer: results of a retrospective, single-center analysis from 2000 to 2005. Anticancer Drugs. 2011; 22:933–9. 10.1097/CAD.0b013e32834860af 21666437

[7] Hurley J, Reis IM, Rodgers SE, Gomez-Fernandez C, Wright J, Leone JP, Larrieu R, Pegram MD. The use of neoadjuvant platinum-based chemotherapy in locally advanced breast cancer that is triple negative: retrospective analysis of 144 patients. Breast Cancer Res Treat. 2013; 138:783–94. 10.1007/s10549-013-2497-y 23542956

[8] Goto W, Kashiwagi S, Takada K, Asano Y, Takahashi K, Fujita H, Takashima T, Tomita S, Hirakawa K, Ohira M. Significance of intrinsic breast cancer subtypes on the long-term prognosis after neoadjuvant chemotherapy. J Transl Med. 2018; 16:307. 10.1186/s12967-018-1679-0 30413161 PMC6230295

[9] Zhu Q, Sheng Y, Li W, Wang J, Ma Y, Du B, Tang Y. Erianin, a novel dibenzyl compound in Dendrobium extract, inhibits bladder cancer cell growth via the mitochondrial apoptosis and JNK pathways. Toxicol Appl Pharmacol. 2019; 371:41–54. 10.1016/j.taap.2019.03.027 30946863

[10] Yang L, Hu Y, Zhou G, Chen Q, Song Z. Erianin suppresses hepatocellular carcinoma cells through down-regulation of PI3K/AKT, p38 and ERK MAPK signaling pathways. Biosci Rep. 2020; 40:BSR20193137. 10.1042/BSR20193137 32677672 PMC7385585

[11] Chen YT, Hsieh MJ, Chen PN, Weng CJ, Yang SF, Lin CW. Erianin Induces Apoptosis and Autophagy in Oral Squamous Cell Carcinoma Cells. Am J Chin Med. 2020; 48:183–200. 10.1142/S0192415X2050010X 31903779

[12] Wang H, Zhang T, Sun W, Wang Z, Zuo D, Zhou Z, Li S, Xu J, Yin F, Hua Y, Cai Z. Erianin induces G2/M-phase arrest, apoptosis, and autophagy via the ROS/JNK signaling pathway in human osteosarcoma cells in vitro and in vivo. Cell Death Dis. 2016; 7:e2247. 10.1038/cddis.2016.138 27253411 PMC5143374

[13] Chen P, Wu Q, Feng J, Yan L, Sun Y, Liu S, Xiang Y, Zhang M, Pan T, Chen X, Duan T, Zhai L, Zhai B, et al. Erianin, a novel dibenzyl compound in Dendrobium extract, inhibits lung cancer cell growth and migration via calcium/calmodulin-dependent ferroptosis. Signal Transduct Target Ther. 2020; 5:51. 10.1038/s41392-020-0149-3 32382060 PMC7205607

[14] Sheng Y, Chen Y, Zeng Z, Wu W, Wang J, Ma Y, Lin Y, Zhang J, Huang Y, Li W, Zhu Q, Wei X, Li S, et al. Identification of Pyruvate Carboxylase as the Cellular Target of Natural Bibenzyls with Potent Anticancer Activity against Hepatocellular Carcinoma via Metabolic Reprogramming. J Med Chem. 2022; 65:460–84. 10.1021/acs.jmedchem.1c01605 34931827

[15] Sun J, Fu X, Wang Y, Liu Y, Zhang Y, Hao T, Hu X. Erianin inhibits the proliferation of T47D cells by inhibiting cell cycles, inducing apoptosis and suppressing migration. Am J Transl Res. 2016; 8:3077–86. 27508028 PMC4969444

[16] Liu Z, Huang L, Sun L, Nie H, Liang Y, Huang J, Wu F, Hu X. Ecust004 Suppresses Breast Cancer Cell Growth, Invasion, and Migration via EMT Regulation. Drug Des Devel Ther. 2021; 15:3451–61. 10.2147/DDDT.S309132 34408399 PMC8364433

[17] Xu Y, Fang R, Shao J, Cai Z. Erianin induces triple-negative breast cancer cells apoptosis by activating PI3K/Akt pathway. Biosci Rep. 2021; 41:BSR20210093. 10.1042/BSR20210093 34036307 PMC8202065

[18] Zhang HQ, Xie XF, Li GM, Chen JR, Li MT, Xu X, Xiong QY, Chen GR, Yin YP, Peng F, Chen Y, Peng C. Erianin inhibits human lung cancer cell growth via PI3K/Akt/mTOR pathway in vitro and in vivo. Phytother Res. 2021; 35:4511–25. 10.1002/ptr.7154 34236105

[19] Luo Q, Li X, Gan G, Yang M, Chen X, Chen F. PPT1 Reduction Contributes to Erianin-Induced Growth Inhibition in Oral Squamous Carcinoma Cells. Front Cell Dev Biol. 2021; 9:764263. 10.3389/fcell.2021.764263 35004674 PMC8740138

[20] Yang A, Li MY, Zhang ZH, Wang JY, Xing Y, Ri M, Jin CH, Xu GH, Piao LX, Jin HL, Zuo HX, Ma J, Jin X. Erianin regulates programmed cell death ligand 1 expression and enhances cytotoxic T lymphocyte activity. J Ethnopharmacol. 2021; 273:113598. 10.1016/j.jep.2020.113598 33220359

[21] Wang P, Jia X, Lu B, Huang H, Liu J, Liu X, Wu Q, Hu Y, Li P, Wei H, Liu T, Zhao D, Zhang L, et al. Erianin suppresses constitutive activation of MAPK signaling pathway by inhibition of CRAF and MEK1/2. Signal Transduct Target Ther. 2023; 8:96. 10.1038/s41392-023-01329-3 36872366 PMC9986241

[22] Sun Y, Li G, Zhou Q, Shao D, Lv J, Zhou J. Dual Targeting of Cell Growth and Phagocytosis by Erianin for Human Colorectal Cancer. Drug Des Devel Ther. 2020; 14:3301–13. 10.2147/DDDT.S259006 32848368 PMC7429191

[23] Miao Q, Deng WQ, Lyu WY, Sun ZT, Fan SR, Qi M, Qiu SH, Zhu YR, Lin JP, Chen MF, Deng LJ. Erianin inhibits the growth and metastasis through autophagy-dependent ferroptosis in KRAS(G13D) colorectal cancer. Free Radic Biol Med. 2023; 204:301–12. 10.1016/j.freeradbiomed.2023.05.008 37217090

[24] Liu Z, Guo F, Wang Y, Li C, Zhang X, Li H, Diao L, Gu J, Wang W, Li D, He F. BATMAN-TCM: a Bioinformatics Analysis Tool for Molecular mechANism of Traditional Chinese Medicine. Sci Rep. 2016; 6:21146. 10.1038/srep21146 26879404 PMC4754750

[25] Wang X, Shen Y, Wang S, Li S, Zhang W, Liu X, Lai L, Pei J, Li H. PharmMapper 2017 update: a web server for potential drug target identification with a comprehensive target pharmacophore database. Nucleic Acids Res. 2017; 45:W356–60. 10.1093/nar/gkx374 28472422 PMC5793840

[26] Yao ZJ, Dong J, Che YJ, Zhu MF, Wen M, Wang NN, Wang S, Lu AP, Cao DS. TargetNet: a web service for predicting potential drug-target interaction profiling via multi-target SAR models. J Comput Aided Mol Des. 2016; 30:413–24. 10.1007/s10822-016-9915-2 27167132

[27] Davis AP, Wiegers TC, Wiegers J, Wyatt B, Johnson RJ, Sciaky D, Barkalow F, Strong M, Planchart A, Mattingly CJ. CTD tetramers: a new online tool that computationally links curated chemicals, genes, phenotypes, and diseases to inform molecular mechanisms for environmental health. Toxicol Sci. 2023; 195:155–68. 10.1093/toxsci/kfad069 37486259 PMC10535784

[28] Queralt-Rosinach N, Piñero J, Bravo À, Sanz F, Furlong LI. DisGeNET-RDF: harnessing the innovative power of the Semantic Web to explore the genetic basis of diseases. Bioinformatics. 2016; 32:2236–8. 10.1093/bioinformatics/btw214 27153650 PMC4937199

[29] Fang T, Liu L, Liu W. Network pharmacology-based strategy for predicting therapy targets of Tripterygium wilfordii on acute myeloid leukemia. Medicine (Baltimore). 2020; 99:e23546. 10.1097/MD.0000000000023546 33327305 PMC7738111

[30] The Gene Ontology Consortium. Expansion of the Gene Ontology knowledgebase and resources. Nucleic Acids Res. 2017; 45:D331–8. 10.1093/nar/gkw1108 27899567 PMC5210579

[31] Kanehisa M, Furumichi M, Tanabe M, Sato Y, Morishima K. KEGG: new perspectives on genomes, pathways, diseases and drugs. Nucleic Acids Res. 2017; 45:D353–61. 10.1093/nar/gkw1092 27899662 PMC5210567

[32] Meng X, Yu X, Liu C, Wang Y, Song F, Huan C, Huo W, Zhang S, Li Z, Zhang J, Zhang W, Yu J. Effect of ingredients from Chinese herbs on enterovirus D68 production. Phytother Res. 2019; 33:174–86. 10.1002/ptr.6214 30346067

[33] Zhang T, Ouyang H, Mei X, Lu B, Yu Z, Chen K, Wang Z, Ji L. Erianin alleviates diabetic retinopathy by reducing retinal inflammation initiated by microglial cells via inhibiting hyperglycemia-mediated ERK1/2-NF-κB signaling pathway. FASEB J. 2019; 33:11776–90. 10.1096/fj.201802614RRR 31365278 PMC6902687

[34] Su C, Zhang P, Liu J, Cao Y. Erianin inhibits indoleamine 2, 3-dioxygenase -induced tumor angiogenesis. Biomed Pharmacother. 2017; 88:521–8. 10.1016/j.biopha.2017.01.090 28129624

[35] Yu Z, Zhang T, Gong C, Sheng Y, Lu B, Zhou L, Ji L, Wang Z. Erianin inhibits high glucose-induced retinal angiogenesis via blocking ERK1/2-regulated HIF-1α-VEGF/VEGFR2 signaling pathway. Sci Rep. 2016; 6:34306. 10.1038/srep34306 27678303 PMC5039671

[36] Dong H, Wang M, Chang C, Sun M, Yang F, Li L, Feng M, Zhang L, Li Q, Zhu Y, Qiao Y, Xie T, Chen J. Erianin inhibits the oncogenic properties of hepatocellular carcinoma via inducing DNA damage and aberrant mitosis. Biochem Pharmacol. 2020; 182:114266. 10.1016/j.bcp.2020.114266 33035506

[37] Liu YT, Hsieh MJ, Lin JT, Chen G, Lin CC, Lo YS, Chuang YC, Hsi YT, Chen MK, Chou MC. Erianin induces cell apoptosis through ERK pathway in human nasopharyngeal carcinoma. Biomed Pharmacother. 2019; 111:262–9. 10.1016/j.biopha.2018.12.081 30590314

[38] Li M, He Y, Peng C, Xie X, Hu G. Erianin inhibits human cervical cancer cell through regulation of tumor protein p53 via the extracellular signal-regulated kinase signaling pathway. Oncol Lett. 2018; 16:5006–12. 10.3892/ol.2018.9267 30250566 PMC6144433

[39] Chen F, Wang Q, Yu X, Yang N, Wang Y, Zeng Y, Zheng Z, Zhou F, Zhou Y. MCPIP1-mediated NFIC alternative splicing inhibits proliferation of triple-negative breast cancer via cyclin D1-Rb-E2F1 axis. Cell Death Dis. 2021; 12:370. 10.1038/s41419-021-03661-4 33824311 PMC8024338

[40] Bai X, Ali A, Lv Z, Wang N, Zhao X, Hao H, Zhang Y, Rahman FU. Platinum complexes inhibit HER-2 enriched and triple-negative breast cancer cells metabolism to suppress growth, stemness and migration by targeting PKM/LDHA and CCND1/BCL2/ATG3 signaling pathways. Eur J Med Chem. 2021; 224:113689. 10.1016/j.ejmech.2021.113689 34293698

[41] Yang SJ, Wang DD, Zhong SL, Chen WQ, Wang FL, Zhang J, Xu WX, Xu D, Zhang Q, Li J, Zhang HD, Hou JC, Mao L, Tang JH. Tumor-derived exosomal circPSMA1 facilitates the tumorigenesis, metastasis, and migration in triple-negative breast cancer (TNBC) through miR-637/Akt1/β-catenin (cyclin D1) axis. Cell Death Dis. 2021; 12:420. 10.1038/s41419-021-03680-1 33911067 PMC8080849

[42] Camorani S, Passariello M, Agnello L, Esposito S, Collina F, Cantile M, Di Bonito M, Ulasov IV, Fedele M, Zannetti A, De Lorenzo C, Cerchia L. Aptamer targeted therapy potentiates immune checkpoint blockade in triple-negative breast cancer. J Exp Clin Cancer Res. 2020; 39:180. 10.1186/s13046-020-01694-9 32892748 PMC7487859

[43] Camorani S, Hill BS, Collina F, Gargiulo S, Napolitano M, Cantile M, Di Bonito M, Botti G, Fedele M, Zannetti A, Cerchia L. Targeted imaging and inhibition of triple-negative breast cancer metastases by a PDGFRβ aptamer. Theranostics. 2018; 8:5178–99. 10.7150/thno.27798 30429893 PMC6217067

[44] Lu Y, Yang G, Xiao Y, Zhang T, Su F, Chang R, Ling X, Bai Y. Upregulated cyclins may be novel genes for triple-negative breast cancer based on bioinformatic analysis. Breast Cancer. 2020; 27:903–11. 10.1007/s12282-020-01086-z 32338339

[45] Novitasari D, Jenie RI, Kato JY, Meiyanto E. The integrative bioinformatic analysis deciphers the predicted molecular target gene and pathway from curcumin derivative CCA-1.1 against triple-negative breast cancer (TNBC). J Egypt Natl Canc Inst. 2021; 33:19. 10.1186/s43046-021-00077-1 34337682 PMC13316920

[46] Huang S, Hu P, Lakowski TM. Bioinformatics driven discovery of small molecule compounds that modulate the FOXM1 and PPARA pathway activities in breast cancer. Pharmacogenomics J. 2023; 23:61–72. 10.1038/s41397-022-00297-1 36424525 PMC10382320

[47] Goode G, Gunda V, Chaika NV, Purohit V, Yu F, Singh PK. MUC1 facilitates metabolomic reprogramming in triple-negative breast cancer. PLoS One. 2017; 12:e0176820. 10.1371/journal.pone.0176820 28464016 PMC5413086

[48] Zhou R, Yazdanifar M, Roy LD, Whilding LM, Gavrill A, Maher J, Mukherjee P. CAR T Cells Targeting the Tumor MUC1 Glycoprotein Reduce Triple-Negative Breast Cancer Growth. Front Immunol. 2019; 10:1149. 10.3389/fimmu.2019.01149 31178870 PMC6543840

[49] Narrandes S, Huang S, Murphy L, Xu W. The exploration of contrasting pathways in Triple Negative Breast Cancer (TNBC). BMC Cancer. 2018; 18:22. 10.1186/s12885-017-3939-4 29301506 PMC5753474

[50] Blücher C, Iberl S, Schwagarus N, Müller S, Liebisch G, Höring M, Hidrobo MS, Ecker J, Spindler N, Dietrich A, Burkhardt R, Stadler SC. Secreted Factors from Adipose Tissue Reprogram Tumor Lipid Metabolism and Induce Motility by Modulating PPARα/ANGPTL4 and FAK. Mol Cancer Res. 2020; 18:1849–62. 10.1158/1541-7786.MCR-19-1223 32859692

[51] Apaya MK, Hsiao PW, Yang YC, Shyur LF. Deregulating the CYP2C19/Epoxy-Eicosatrienoic Acid-Associated FABP4/FABP5 Signaling Network as a Therapeutic Approach for Metastatic Triple-Negative Breast Cancer. Cancers (Basel). 2020; 12:199. 10.3390/cancers12010199 31941087 PMC7016875

[52] Kwong SC, Jamil AHA, Rhodes A, Taib NA, Chung I. Metabolic role of fatty acid binding protein 7 in mediating triple-negative breast cancer cell death via PPAR-α signaling. J Lipid Res. 2019; 60:1807–17. 10.1194/jlr.M092379 31484694 PMC6824484

[53] Li T, Zhang Q, Zhang J, Yang G, Shao Z, Luo J, Fan M, Ni C, Wu Z, Hu X. Fenofibrate induces apoptosis of triple-negative breast cancer cells via activation of NF-κB pathway. BMC Cancer. 2014; 14:96. 10.1186/1471-2407-14-96 24529079 PMC4015735

[54] Martini M, De Santis MC, Braccini L, Gulluni F, Hirsch E. PI3K/AKT signaling pathway and cancer: an updated review. Ann Med. 2014; 46:372–83. 10.3109/07853890.2014.912836 24897931

[55] Wang Y, Chu F, Lin J, Li Y, Johnson N, Zhang J, Gai C, Su Z, Cheng H, Wang L, Ding X. Erianin, the main active ingredient of Dendrobium chrysotoxum Lindl, inhibits precancerous lesions of gastric cancer (PLGC) through suppression of the HRAS-PI3K-AKT signaling pathway as revealed by network pharmacology and in vitro experimental verification. J Ethnopharmacol. 2021; 279:114399. 10.1016/j.jep.2021.114399 34246740

